# Practical Approach for High-Resolution Airport Pavement Inspection with the Yakumo Multistatic Array Ground-Penetrating Radar System

**DOI:** 10.3390/s18082684

**Published:** 2018-08-15

**Authors:** Li Yi, Lilong Zou, Motoyuki Sato

**Affiliations:** 1 Fukushima Renewable Energy Institute, National Institute of Advanced Industrial Science and Technology, Koriyama 963-0298, Japan; 2 Affiliation Center for Northeast Asian Studies, Tohoku University, Sendai 980-8576, Japan; zou_ll@cneas.tohoku.ac.jp (L.Z.); motoyuki.sato.b3@tohoku.ac.jp (M.S.)

**Keywords:** ground-penetrating radar (GPR), multistatic radar, common midpoint (CMP), velocity estimation

## Abstract

It is important to identify the thin cracks within the airport pavement layers. To achieve this goal, a practical interferometric approach using the Yakumo multistatic ground-penetrating radar system was developed to detect the slight variability in wave propagation velocity and/or thickness caused by the thin cracks. In comparison with the conventional common midpoint (CMP) velocity estimation method, the proposed method can provide much higher-resolution estimations of slight deviations in the velocity and thickness from their corresponding reference values in the undamaged asphalt through the comparison of two CMP datasets. These deviations can be obtained analytically instead of graphically extracted from the CMP velocity spectrum. The proposed approach was not only analyzed using the simulated datasets, but also practically demonstrated at both an experimental model site and an actual airport site. In the simulation tests, velocity deviations on the order of a few millimeters per nanosecond were detected, and the experimental results shows good agreement with the ground truth and coring samples. This method provides a novel way to inspect partially damaged pavement when the thin cracks are difficult to detect using the reflected signals.

## 1. Introduction

The maintenance of runways and taxiways is an important task in ensuring the safety of airport facilities. The airport pavement must be constructed with high standards which include perfect flatness, toughness and uniformity, hence multiple layers with different materials are required [1]. However, such multi-layer structures is not perfect to prevent all the problems, especially due to the minor spaces left between different layers. With the deterioration of the pavement layer, high temperatures and pressures produced by the aircraft landing on runways can cause small drops of water present within the minor spaces to evaporate and extend to thin cracks. When these thin cracks extended enough to affect the pavement integrity, the pavement may break, which leads to distortions or cracks appearing on the surface [1,2]. Hence, it is important to identify these potentially damaged pavements and repair them before they fail. In Japan, airport pavements have conventionally been inspected by skilled workers based on the echoes produced by striking the pavement, tests that must be conducted every night because of their low efficiency and accuracy. Therefore, a better non-destructive inspection (NDI) method is required.

NDI methods applied for pavement inspection application have been reported by [3,4,5,6]. Among the different methods, ground-penetrating radar (GPR) is known to have the best resolution among them and is widely used for civil engineering applications [4,5,6,7,8,9,10,11]. However, due to the limitation of its penetrating depth and resolution, GPR is mainly used for detecting large-scale voids or distortions for subsurface inspection [5,6], while the main target in the inspection of airport roadways are thin cracks within the asphalt layer whose apertures may be on the order of millimeters which is far beyond the resolution of GPR with a few GHz center frequency. Hence it is difficult to observe the reflections from the thin cracks directly, as pointed out in [9,10].

Although the thin cracks are difficult to detect directly, the density variation of the pavement is shown to be an indicator for pavement inspection due to its high uniformity and it has achieved with a variety of methods [4]. Nuclear density gauges and ultrasonic measurement have excellent accuracy in laboratory experiments, but they are not suitable for large scale surveys [11,12,13]; infrared tomography shows good potential for large scale inspection, but it is strongly affected by the observation conditions such as ambient temperature, wind speed, and sky conditions, which make it difficult to use for real applications [4,14].

Based on the same idea, it is also possible to inspect the pavement by estimating the slight spatial variability in the permittivity caused by the formation of these cracks [4,15,16]. From the perspective of inversion methods with GPR, it has been reported that the estimated permittivity can be used as an important indicator of the presence of subsurface anomalies [16]. The common-mid point (CMP) GPR antenna array allows for accurate estimation of the permittivity without resorting to complex processing and inversion techniques. This approach has proved to be promising for subsurface characterization applications such as soil moisture monitoring or pavement thickness estimations [17,18,19]. However, the relatively low resolution of the permittivity estimation and the difficulties associated with acquiring of large-scale CMP data are the main problems with this technique that make it difficult to apply for our purpose.

To overcome these problems, the use of the Yakumo multistatic GPR system [8] for the acquisition of large-scale CMP datasets is proposed in this paper. Some limitations and features of conventional CMP velocity analysis were highlighted. Based on the conventional CMP method, a novel method in combination with an interferometric approach of analyzing two CMP datasets was developed in this study. The proposed method can be used to evaluate deviations in the depth and velocity with high precision using much simpler calculations than in the conventional method. Through simulated data and actual experiments, it was demonstrated that the proposed method shows good performance in detecting slight deviations in the depth and velocity of the upper asphalt layer.

## 2. CMP Velocity Analysis with the Yakumo Multistatic GPR System 

**Figure 1:**
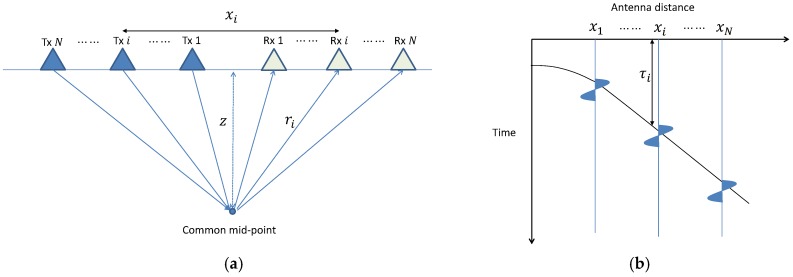
Configuration used to obtain a CMP dataset. (**a**) Data acquisition. (**b**) Corresponding CMP data.

CMP data are unique datasets that can be acquired using a bistatic or multistatic GPR system. To conduct CMP analysis at a fixed measurement position, the signals reflected from a CMP must be measured at positions on both sides of the CMP with different offsets, as shown in Figure 1.

The antenna offset is defined as the distance between the transmitting and receiving antennas constituting an antenna pair. The root mean square (RMS) velocity of the electromagnetic wave propagation within different subsurface layers can be estimated from the velocity spectrum which is obtained with the CMP dataset. Under the assumption that the subsurface medium is homogeneous and horizontally layered, the travel time τ of the reflection signal is given by:

(1) $$\tau \left(x_{i},z,v\right)=\frac{r_{i}}{v}=\frac{\sqrt{x_{i}^{2}+4z^{2}}}{v},$$

where *z* is the depth of the horizontal reflector or the pavement thickness in our case, $x_{i}$ is the antenna offset of the *i*-th trace, *v* is the trial velocity, and $r_{i}$ is the propagation path between the corresponding pair of antennas.

With the acquired CMP dataset *F*, the velocity spectrum *P* can be acquired as:

(2) $$P\left(z,v\right)=\overset{N}{\underset{i=1}{\sum }}F\left(x_{i},\tau_{i}\left(x_{i},z,v\right)\right),$$

which gives the stacked amplitude of the reflected signals at offset $x_{i}$ and travel time $\tau_{i}$ with assumed depth *z* and the trial velocity *v* as it is shown in Figure 1b. When a trial velocity is close to the correct RMS velocity, the stacked energy will be enhanced, and thus the velocity may be estimated from the velocity spectrum where the velocity corresponding to the maximum energy. However, the CMP measurement process with a conventional GPR system is time-consuming and complicated because the two antennas must be manually operated simultaneously.

**Figure 2:**
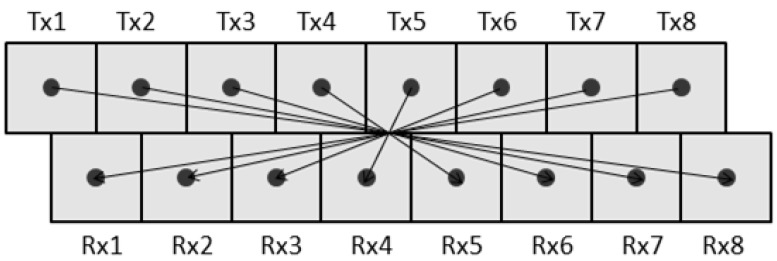
Antenna configuration of the Yakumo system.

To simplify the acquisition of three-dimensional (3D) GPR and CMP data, our group developed a new array system called Yakumo in 2013 [8]. Yakumo is 2 m wide and has eight transmitting and eight receiving antennas, as shown in Figure 2. It is a stepped-frequency continuous-wave (SFCW) radar system that can be operated between 50 MHz and 1.5 GHz. All the transmitters and receivers can be switched sequentially, so traces with any of the 8 × 8 antenna combinations can be acquired. After the data have been acquired along the survey line, a 3D data cube can be generated by synthetic-aperture radar (SAR) processing [8]. With this antenna configuration, it is possible to acquire CMP data at a fixed position, as shown in Figure 2. To enhance the efficiency of the data acquisition, less antenna pairs can be selected to acquire multi parallel CMP data at same time. For example, three parallel CMP datasets can be acquired with six of eight antenna pairs. In comparison with the configurations of common CMP measurement systems, the CMP data acquisition setup of the Yakumo system is very fast and convenient. However, CMP datasets acquired by the Yakumo system include only a few traces, also the antenna offsets of different antenna pairs are not unique.

Theoretically, the exact thickness and propagation velocity within the medium can be determined uniquely. In previous work we have shown that the thin cracks on the order of millimeters filled with air/water will cause small variations in the permittivity [19]. Since the reflections from the thin cracks are difficult to observe directly, it is possible to detect these thin cracks with these small variations. However, the resolution of the velocity spectrum is dependent on the length of the CMP survey line, the wavelength of the signal, the noise level, and the trial velocity update step, which is similar to the SAR imaging procedure [20]. Because of the abovementioned limitations, the energy on velocity spectrum cannot be perfectly focused to a single point hence it may not be sufficient to precisely estimate the permittivity variations.

**Figure 3:**
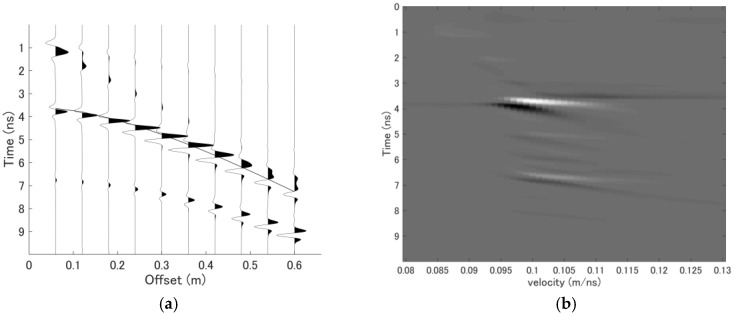
Simulated single-layer CMP dataset and its velocity spectrum. (**a**) CMP dataset for a layer with a thickness of 0.16 m and a propagation velocity of 0.1 m/ns. (**b**) Corresponding velocity spectrum acquired using the stacking method.

As an illustrative example, a simple CMP dataset simulated using the finite-difference time-domain (FDTD) method for one reflection layer with a thickness of 0.16 m and a propagation velocity of 0.1 m/ns is shown in Figure 3a, and the corresponding velocity spectrum is shown in Figure 3b. The propagation velocity and thickness of the reflection layer can be estimated from the velocity spectrum shown in Figure 3b. In Figure 3a, the theoretical arrival time is indicated with a solid line; when this arrival time passes through the peak of each signal trace, the stacked energy will be maximized. However, with the abovementioned problems, it is difficult to pick the accurate velocity in this velocity spectrum. 

On the other hand, because of the different incidence angles and other factors such as dispersion and attenuation, the waveforms at different traces will be slightly distorted, hence errors in the velocity spectrum cannot be prevented. From the perspective of hardware, the resolution can be enhanced by increasing the center frequency, widening the frequency bandwidth, and lengthening the observation lines; however, the increased incident angle or reduced penetrating depth may produce new problems when modifying the system. For example, depending on the thickness of the pavement layer, the maximum antenna offset is limited due to the incident angle. For the Yakumo system, it is found that when the antenna offset is larger than one meter the reflected signal will become almost invisible. Hence only six of eight antenna pairs are applied in later experiments.

In a previous study, we proposed another approach combining the compressed sensing (CS) method with the conventional velocity analysis method [19]. This approach can provide a high-resolution velocity spectrum by using inversion approach to reduce the effect of waveform and spatially sparse sampling. However, the performance of the CS-based algorithm is not always robust and the iterative calculation is time-consuming. While for the new approach introduced in above, the effect of waveform is canceled by introducing the time delay and the artifact problem in velocity spectrum is prevented which also lead to fast and robust calculation. Thus, CS-based the solution is more accurate mathematically but not physically.

## 3. Estimation of Deviations in Layer Velocity and Thickness by Subsample Time Delay Estimation

As mentioned above, to enhance the results used to evaluate slight spatial variability in the propagation velocity and thickness of the asphalt layer, it is critical to find a high-resolution method to reduce the effect of waveform distortions. On the other hand, graphical extraction from the velocity spectrum should be simplified by obtaining the numerical results directly. Since the pavement layer is a relatively constant target, an interferometric method may be a good solution to this problem.

Although it is difficult to accurately measure the travel time of the reflected signal, it is relatively simple to estimate the time delay between each antenna pair of two CMP datasets. The time delay with different antenna offsets can be acquired with the cross-correlation method. For conventional CMP method, waveforms obtained with different antenna pairs may vary because of the different paths and angles of incidence, but waveforms obtained with same antenna pair at different positions are much more robust against variation. Hence, the accuracy of deviation estimation can be greatly enhanced.

Once the time delay has been acquired, it is assumed that there exist slight deviations Δv and Δz in the propagation velocity and layer thickness from their reference values *v* and *z* estimated at reference CMP dataset. These variations are considered to satisfy:

(3) $$\frac{\Delta z}{z},\;\frac{\Delta v}{v}\ll 1,$$

On the basis of this assumption, the first-order differential arrival time can be derived as:

(4) $$\Delta \tau \left(x_{i}\right)=\Delta \left(\frac{1}{v}\sqrt{4z^{2}+x_{i}{}^{2}}\right)=\frac{4z}{v}{\left(\sqrt{4z^{2}+x_{i}{}^{2}}\right)}^{-1}\Delta z-\frac{1}{v^{2}}\sqrt{4z^{2}+x_{i}{}^{2}}\Delta v.$$

This indicates that if the velocity and layer thickness are known at a certain position, hereafter referred to as the reference point. The propagation velocity and thickness at reference point can be either estimated with conventional CMP method at a known healthy pavement region or using the average value of the inspected region. Then the slight deviations Δ*v* and Δ*z* in the propagation velocity and thickness from the reference values can be estimated at an arbitrary measurement position, hereafter referred to the monitoring point, from the time delay Δτ at an antenna offset of $x_{i}$ by using the CMP dataset. For practical application, on the basis of the known velocity and thickness at the reference point, a time window with about 1–2 wavelength wide should be applied to filter out other irrelevant reflections since the proposed method only use the time delay of the reflected signals between two CMP dataset. Since the white noises can be canceled with the cross-correlation process, the proposed method is less affected by the strong noise in theory.

At least two antenna pairs are necessary to solve Equation (4), and it can be solved as an overdetermined problem by using the method of least squares. This yields a result similar to that obtained using the CMP velocity analysis method of estimating deviations in the propagation velocity and thickness from the time delay with different antenna pairs. Although Equation (4) is a simple differential method of estimating the slight deviations in the propagation velocity and thickness, the uncertainty of overdetermined problem makes the propagation velocity and thickness difficult to be solved perfectly, especially when small number of antenna pairs are used. However, for asphalt inspection applications, the detection of anomalies is more important than accurately modeling the accurate velocity and thickness values.

**Figure 4:**
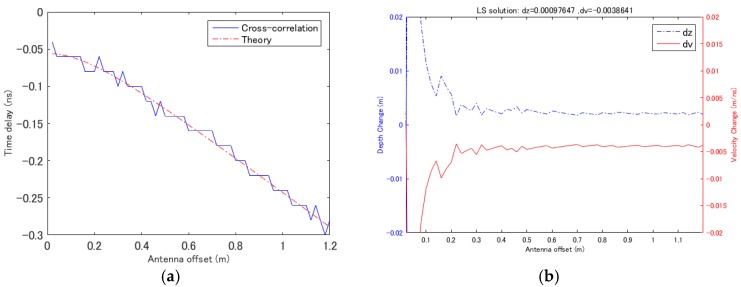
Simultaneous estimation of deviations in the propagation velocity and thickness with simulated data acquired by ray tracing. (**a**) Time delay estimation using the cross-correlation method. (**b**) Estimation results obtained using the proposed method with two antenna pairs; one with 0.05 m offset and all other antenna pairs. The least squares solution is given at the top of the figure.

A simple example of the proposed estimation method is shown in Figure 4. The simulated data were generated by ray tracing and convoluted with a Ricker wavelet with a center frequency of 500 MHz. The reference data were assumed to be for a medium with a thickness of 15 cm and a propagation velocity of 0.1 m/ns, and thickness and velocity deviations of Δ*z* = 1 mm and Δ*v* = 0.004 m/ns, respectively, were added to the simulated data at the monitoring point. Because of the simplicity of the model, the theoretical time delay agreed well with that estimated using the cross-correlation method, as shown in Figure 4a. For this simple case, although a dense dataset is simulated with 0.05 m offset, it is possible to calculate the deviations in the velocity and thickness by using less antenna pairs. The minimum two antenna pairs, one with 0.05 m offset and all other offsets are selected for calculation and the result is shown in Figure 4b. The least squares solution with all antenna pairs was also calculated, and it yielded a thickness deviation Δ*z* of 0.97 mm and a velocity deviation Δ*v* of 0.0038 m/ns.

As shown in Figure 4b, most of the antenna combinations yield accurate estimations, but when the antennas in a pair are close to one another, the results become inaccurate. This is caused by the small difference between the time delays of two nearby antennas. This indicates that this method does not require many antenna pairs but does require sufficiently large time delay differences, which means larger antenna offsets are necessary. As has been noted by Yilmaz [7], near-offset traces are more sensitive to deviations in thickness, whereas far-offset traces are more sensitive to deviations in propagation velocity due to the effect of waveform distortion. With the purposed method such effect can be reduced and it is demonstrated that accurate results can be given with two pair of antennas of more than 0.15 m offset. However, due to the uncertainty of the field dataset, efficient number of the antenna pairs are required to enhance the accuracy of the calculation. For the field experiments demonstrated in later sections, six of eight antenna pairs of Yakumo system is applied for calculation, and it is found that constant least square results can be achieved by using four to six antenna pairs, and suspicions results frequently appears when less than four pairs of antennas are used for calculation.

The previous example can be achieved with only two antenna pairs because of the precision of the time delay estimation and simple modeling which is not realistic for fieldwork. For FDTD-simulated data and real data, the time delay estimation is more difficult due to the effect noise and attenuation, and an advanced time delay estimation method is necessary in these cases. 

To enhance the accuracy of the time delay estimation, an iterative cross-correlation approach is introduced for subsample time delay estimation in [21]. The proposed method is based on the Nyquist–Shannon sampling theorem, and it is applied by iteratively calculating the cross-correlation of two signals. The discrete frequency-domain cross-correlation function can be calculated as:

(5) XC(n)=S1(n)S2(n),

where XC, *S*1, and *S*2 are the discrete Fourier transforms of the discrete time-domain signals *xc*, *s*1, and *s*2, respectively and n is discrete frequency point. The continuous cross-correlation function $\tilde{xc}$ can then be calculated as:

(6) $$\tilde{xc}\left(t\right)=XC\left(0\right)+\overset{\frac{N}{2}-2}{\underset{n=1}{\sum }}2XC\left(n\right)e^{\frac{2\pi i}{K}tn},$$

where *K* is the number of sampling points in the discrete time-domain signal. The subsample time delay *d* can then be estimated by optimizing $\tilde{xc}$ as:

(7) $$d=\mathrm{argmax}\left(\tilde{xc}\left(t\right)\right).$$

Because no closed-form solution to this equation exists, numerical methods must be used to maximize $\tilde{xc}$. Here, a revised version of the method introduced by Nentwig [22] was applied to solve (7). It is also noted that the iterative method shows better performance when there are slight differences between the phases and amplitudes of the two correlated signals, which makes it robust against the waveform differences caused by slight deviations in the propagation velocity and thickness. 

**Figure 5:**
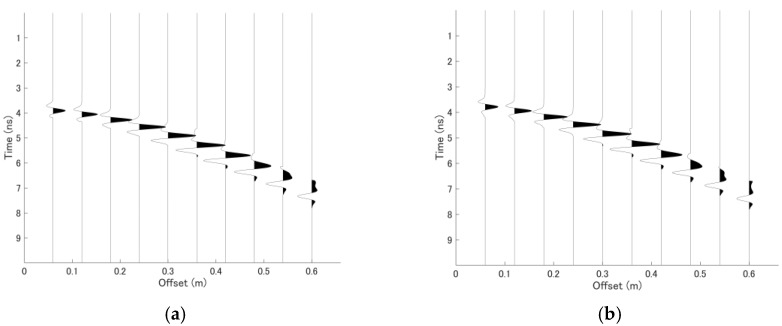
FDTD simulation of two single-layer CMP datasets with slight differences in velocity and thickness. (**a**) CMP dataset obtained at the reference point with a thickness of 15 cm and a velocity of 0.1 m/ns. (**b**) CMP dataset obtained at the monitoring point with thickness and velocity deviations of 1 cm and 0.03 m/ns, respectively, with respect to the reference values.

An FDTD simulation example was then conducted. A Ricker wavelet with a center frequency of 500 MHz was used for the simulation. The reference data were assumed to correspond to a depth of 15 cm and a propagation velocity of 0.1 m/ns with a thickness deviation of 1 cm and a velocity deviation of 0.003 m/ns added to the simulated data as a monitoring point. Additionally, an 8 mm air gap was included between the antenna and the surface. In this example, a much larger thickness deviation was applied than in the previous simple example to demonstrate the sensitivity of the proposed approach to relatively large thickness variation. This simulation represents a situation in which variation in the thickness of the asphalt layer is introduced during construction, as the proposed method should be able to distinguish between the influence of thickness deviations and that of velocity deviations. The CMP datasets obtained at the reference and monitoring points are shown in Figure 5. The observation length used in this example was 0.6 m, and the offset interval was 6 cm. It should be noted that slight waveform variations appear due to the effect of the thin air-layer within the asphalt layer, especially in far-offset traces. 

**Figure 6:**
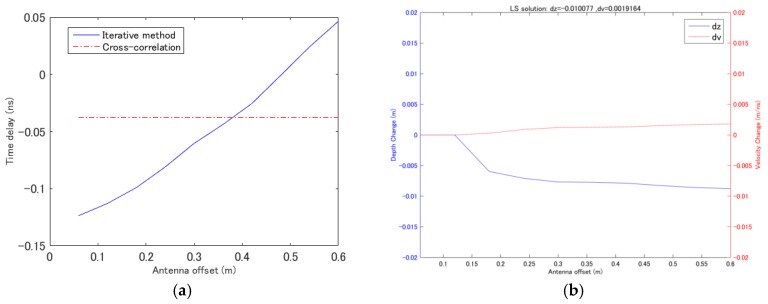
Simultaneous estimation of deviations in the propagation velocity and thickness with simulated data acquired using the FDTD method. (**a**) Time delay estimation using the cross-correlation method and the proposed iterative method. (**b**) Estimation results obtained using the proposed method with two antenna pairs; one with 0.05 m offset and all other antenna pairs. The least squares solution is given at the top of the figure.

The estimated time delay and similar plotting results with an antenna offset interval of 0.6 m are shown in Figure 6. In this simulation scenario, the conventional cross-correlation method failed to estimate the time delay changes, whereas with the iterative method, the time delay curve could be recovered well. The least squares solution was calculated with all of the antenna pairs, and the thickness and velocity deviations were obtained as Δ*z* = 1 cm and Δ*v* = 0.0019 m/ns, respectively. The least squares solution for the velocity deviation was slightly different from the actual value of 0.003 m/ns. Although these results are not precisely correct, this method shows good performance in recognizing deviations in the thickness and/or propagation velocity from their corresponding reference values. There are two main reasons for the error in the results. First, as mentioned previously, for a signal simulated using the FDTD method, the waveform is not constant if the properties of the estimated layer are changed. The slight deviations in the permittivity and thickness from their corresponding reference values cause the waveform to change slightly; hence, the estimated time delay may not be completely accurate. Second, the presence of a layer of air between the antenna and the ground surface causes the RMS velocity of the asphalt layer slightly changed. This is also a problem when analyzing actual data using this method; the influence of the inhomogeneity of the asphalt layer together with the gap between the antenna and the surface may cause the actual arrival time curve to deviate from the perfect hyperbolic curve predicted by the theory, hence the errors will be introduced to analytic results.

## 4. Field Experiments

**Figure 7:**
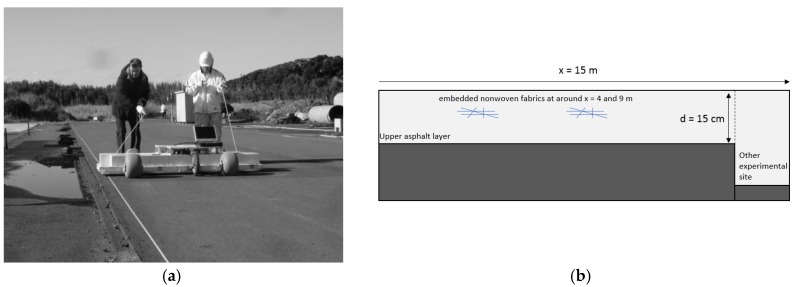
Data acquisition with Yakumo on the airport runway model at the Nobi experiment site. (**a**) Experiment site. (**b**) Pavement structure.

The first experiment site was an airport taxiway model at the Port and Airport Research Institute in Nobi (Kanagawa Prefecture, Japan). A photograph of the experiment site is shown in Figure 7a, and a 15-m-long survey line was selected for testing.

The taxiway model contains two manmade fracture zones created by embedding nonwoven fabrics within the 15-cm-thick asphalt layers, and each of the fracture zones is 0.5 m wide. The fracture zones are located at positions of approximately x = 4 and 9 m along the survey line, as shown in Figure 7b. After the pavement layer was built, a 20-ton truck was used to simulate an airplane landing by moving along the pavement layer repeatedly. Water was also injected into the asphalt layers at the same time to simulate the destruction processing that is introduced before. The aperture of the cracks within the manmade fracture zones was built to be less than one millimeter, but after the site was built and destruction simulation it is difficult to measure them accurately. In most of the profiles, the reflected signals from the cracks were very weak and difficult to distinguish; the survey line discussed here was selected to yield relatively clear signals.

**Figure 8:**
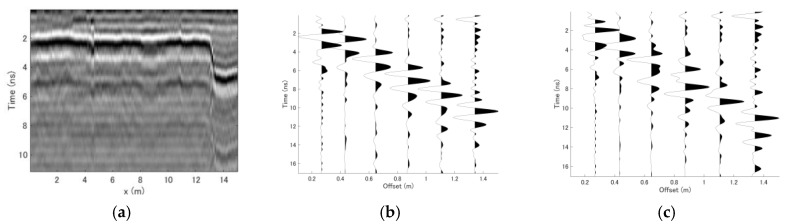
Datasets obtained along the survey line. (**a**) Vertical slice of the survey line obtained using 0.24 m antenna offset. (**b**) CMP dataset at x = 0.5 m. (**c**) CMP dataset at x = 8.5 m, where the manmade fracture zone was detected.

Figure 8a shows the vertical profile obtained along the survey line by using 0.24 m antenna offset, which yields results similar to those that would be obtained using a conventional monostatic GPR system. The first fracture zone at 4 m is observable in the vertical profile, but the other zone at 9 m is not clear. Figure 8b,c show CMP datasets obtained at x = 0.5 and 8.5 m, which correspond to locations of undamaged pavement and pavement with a manmade fracture zone. The time delay can be observed more clearly with far-offset traces, but the waveform distortion in these traces is also more severe. This is the main limitation of the conventional CMP velocity estimation method [7].

**Figure 9:**
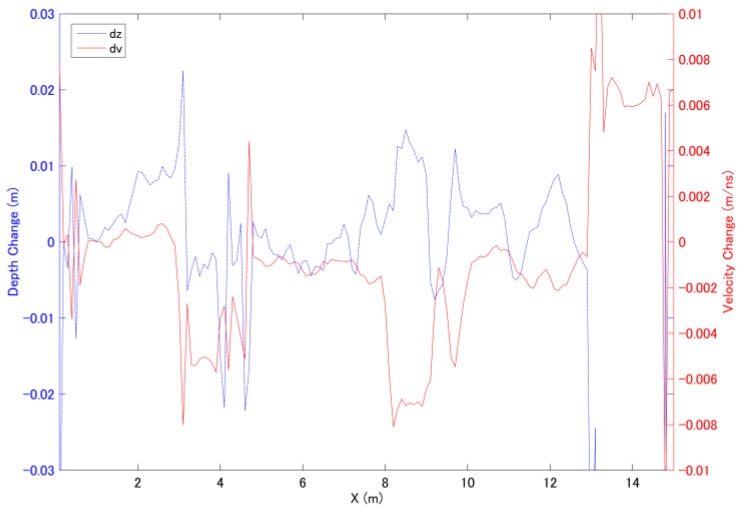
Simultaneous estimations of the velocity and thickness deviations along the survey line. The reference CMP dataset was obtained at x = 0.5 m.

The least squares solutions for Δv and Δz obtained using six antenna pairs (two of the far-offset antennas pairs were not used) are plotted in Figure 9. The thickness and subsurface velocity of the reference CMP dataset were estimated to be 0.16 m and 0.135 m/ns, respectively, using conventional CMP velocity analysis at x = 0.5 m, and the results obtained at one of the monitoring points located on the fracture zone is shown in Figure 9. The manmade fracture zones are clearly observable in the analysis results at approximately 4 and 9 m. It is noticeable that the estimated velocity and thickness values appear to be correlated, and it is known as the crosstalk. When the estimated time delay is less accurate the crosstalk effect will be stronger which means that the effect of velocity or thickness deviation cannot be distinguished at same time. In this example, the velocity deviation is more sensitive than the thickness deviation especially at x = 4 m position.

As was discussed at the end of the previous section, the inhomogeneity of the subsurface is much more complex than that in the simulated datasets; hence, in most cases, the estimated arrival time delay is not ideal since the arrival time curve is not a perfect hyperbolic. It should also be noted that the selection of the reference dataset does not influence the trends of the estimated velocity and thickness deviations along the survey line. However, the numerical result may become inaccurate. In this case, the practical application of this method is not expected to yield an accurate numerical estimation of the deviations in the velocity and thickness. However, the detection of subsurface anomalies which cannot be observed using reflection methods is more important and can be achieved using the proposed method.

**Figure 10:**
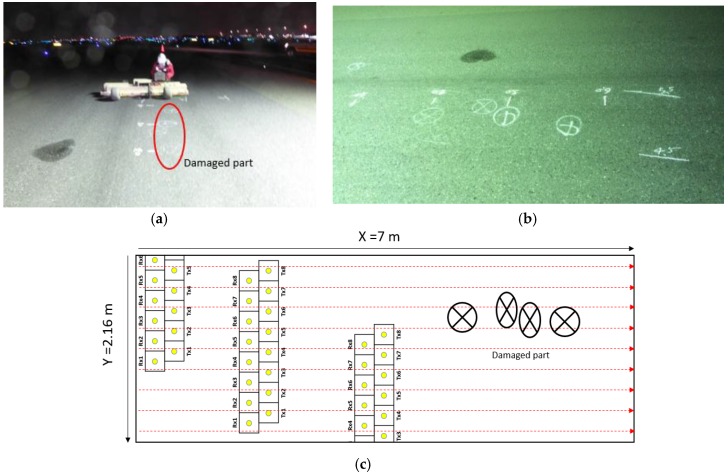
Data acquisition with Yakumo at Haneda international Airport. (**a**) Experiment site. (**b**) enlarged ground surface of damaged part; (**c**) sketch of processed area, each dash line indicates a CMP center.

The second experiment site is Haneda international Airport located in Tokyo, Japan. The experiment site is shown in Figure 10a, and a small test area of dimensions 7 m × 4 m was selected for demonstration. The damaged part located with the impact-echo method is circled in Figure 10a, and this part is enlarged as it is shown in Figure 1b. The test area is spanned by three groups of survey lines in the x-direction with half of the width of the Yakumo system in each scan overlapping that of the previous scan, as shown in the schematic in Figure 10c. In each scan, three CMP survey lines can be extracted from the Yakumo dataset, yielding a total of nine CMP survey lines for analysis, as indicated by the dashed lines in Figure 10c. Because each of the antenna elements has dimensions of 24 cm × 24 cm, the distance between each CMP survey line is 24 cm; hence, the inspected area is 7 m × 2.16 m, as shown in Figure 10c, also the damaged part is plotted at the corresponding location.

**Figure 11:**
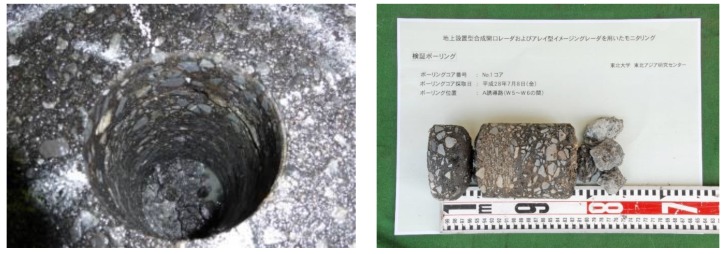
Coring sample acquired from the damaged pavement (x = 6 m) at the Sendai Airport site.

The asphalt layer in the test area is known to be 25 cm thick, and the test area was inspected beforehand by airport staffs by striking the pavement. A damaged region was located at x = 4 to 6 m and y = 0.5 to 1 m, as indicated in Figure 10. To obtain more information on the damaged pavement, validation by coring was also performed after the Yakumo data were acquired. Two coring samples were obtained from within the area indicated in Figure 10, and one of the samples which acquired at x = 6 m is shown in Figure 11. Two thin cracks were observed in this sample. However, the apertures of these cracks in situ were not as large as those shown in Figure 11, because the edges of the cracks were broken during the coring. The apertures of the cracks were evaluated in the middle part of the coring sample and were found to be approximately 1 mm. As mentioned in previously, the reflection from such thin cracks is difficult to observe; with this dataset, no clear reflection from these cracks was observed.

**Figure 12:**
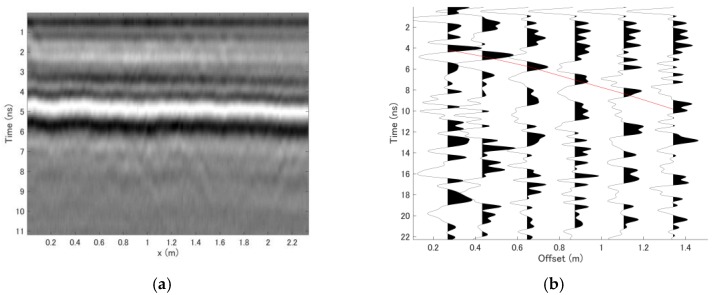
Datasets acquired with Yakumo. (**a**) Vertical profile at y = 0.72 m. (**b**) CMP dataset acquired at x = 2 m and y = 1 m. The solid line indicates the reference thickness and velocity of the reflection layer.

Figure 12a shows the vertical profile at y = 0.72 m; along this line, the pavement is damaged between x = 4 and 6 m. From this vertical profile, no observable reflections were present above the bottom of the asphalt layer at about 5 ns. After examination of the vertical profile, a CMP dataset obtained at x = 2 m and y = 1 m was selected as the reference dataset, and the CMP data obtained in the reference measurement are shown in Figure 12b. The solid line in Figure 12b indicates the known reference thickness and velocity parameters, which were estimated to be 25 cm and 0.148 m/ns, respectively. Here, it should be noted that the CMP data have a different qualitative appearance from those obtained in the previous experiment. This is because the antenna elements of the Yakumo system were oriented perpendicular to one another for its use in other projects. This orientation causes the amplitude to be much weaker than in the previous antenna arrangement, and both the gain function and normalization were applied to this dataset to enhance the far-offset traces.

**Figure 13:**
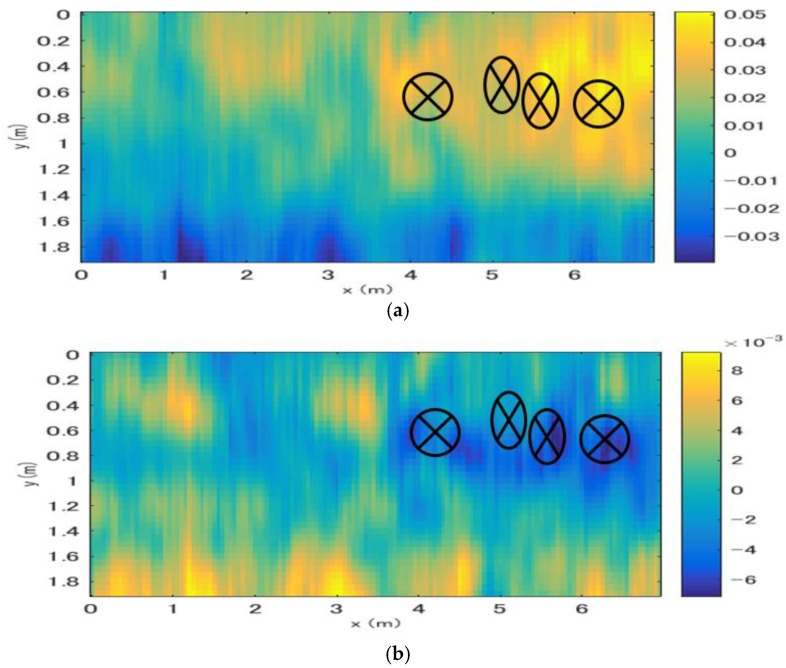
Deviations in the velocity and thickness estimated at the Sendai Airport survey area, the location of damaged parts are also plotted on the results (**a**) Velocity deviations. (**b**) Thickness deviations.

After the entire dataset was processed using the proposed method, the two-dimensional distribution of the velocity and thickness deviations was acquired, as shown in Figure 13. Both the velocity and thickness deviations indicate the presence of damaged pavement between x = 4 and 6 m and y = 0.5 and 1 m; these results agree well with those obtained using the impact-echo method. However, the estimated velocity and thickness deviations reached maximal values of 0.05 m/ns and 8 mm respectively, which are too large by considering the ground truth. The causes of this overestimation have been explained in a previous section; the error is considered to be caused by the distortion of the wavelets by small sources of scatter and the choice of reference point. Hence, it is suggested that this method is best suited for detecting the existence of anomalies in practical applications. The numerical solution should be considered as additional information to be used in conjunction with known parameters at the experiment site.

## 5. Conclusions

In this work, a practical method of shallow pavement inspection using a multistatic GPR system was introduced. The main purpose of this method is the detection of anomalies in a partially damaged asphalt layers containing only thin cracks that are difficult to detect with conventional reflection imaging methods. These thin cracks filled with water and air are considered to produce inhomogeneities in the asphalt layer, and the RMS velocity of signals traveling through the layer at these inhomogeneities differs slightly from that in the undamaged asphalt. Thus, it is expected that such partially damaged regions of the asphalt layer can be detected from these slight deviations in permittivity.

To achieve large-scale subsurface permittivity estimation, a multistatic GPR system called Yakumo has been developed. The special antenna arrangement of the Yakumo system makes it possible to simultaneously acquire several CMP datasets along a survey line. To assess a large number of these CMP datasets with high resolution and good robustness, a method based on conventional CMP velocity analysis combined with an interferometric method was developed in this study. Comparing with the conventional CMP method, this method enables the high-resolution estimation of deviations in the subsurface velocity and thickness from the reference values for the layer structure because it uses the time-delay information instead of travel time which affected by the signal bandwidth and the waveform distortion. On the other hand, it reduces the difficulty of graphically extracting the precise deviations in the velocity and thickness from the velocity spectrum; hence, the processing is greatly simplified in comparison with conventional CMP methods. Theoretically, slight deviations in the velocity and thickness can be detected simultaneously using only a few antenna pairs with different offsets. With various simulation tests and field experiments, it was demonstrated that the proposed method is suitable for locating partially damaged regions in an asphalt layer. The proposed method is more accurate than the conventional CMP velocity estimation method, and the calculation process is much simpler, facilitating its application to real-time inspection.

However, as mentioned previously, this method is still based on the CMP velocity estimation method, which uses curve fitting for single-layer velocity estimations. Although the resolution of this method can be improved using the proposed method, the inhomogeneities detected in the evaluated asphalt layer are considered to alter the RMS velocity in the entire layer; hence, the crosstalk of the estimated velocity and thickness changes cannot be easily prevented. Thus, in practical applications of this method, the estimated deviations in the velocity and thickness should be considered as an indication of the distribution of inhomogeneities within the inspected asphalt layers and not as exact numerical results.
